# Blood levels of microRNAs associated with ischemic heart disease differ between Austrians and Japanese: a pilot study

**DOI:** 10.1038/s41598-020-69332-0

**Published:** 2020-08-12

**Authors:** Ichiro Wakabayashi, Ryoji Eguchi, Yoko Sotoda, Dirk von Lewinski, Harald Sourij, Takashi Daimon, Klaus Groschner, Peter P. Rainer

**Affiliations:** 1 Department of Environmental and Preventive Medicine, Hyogo College of Medicine, Mukogawa-cho 1-1, Nishinomiya, Hyogo 663-8501 Japan; 2 Department of Cardiovascular Surgery, Yamagata Saisei Hospital, Okimachi 79-1, Yamagata, 990-8545 Japan; 3 Division of Cardiology, Medical University of Graz, Auenbruggerplatz 15, 8036 Graz, Austria; 4 Division of Endocrinology and Metabolism, Medical University of Graz, Auenbruggerplatz 15, 8036 Graz, Austria; 5 Department of Biostatistics, Hyogo College of Medicine, Mukogawa-cho 1-1, Nishinomiya, Hyogo 663-8501 Japan; 6 Gottfried Schatz Research Center for Cell Signaling, Metabolism and Aging, Medical University of Graz, Neue Stiftingtalstrasse 6/D04, 8010 Graz, Austria

**Keywords:** Risk factors, Cardiovascular diseases

## Abstract

Mortality from ischemic heart disease (IHD) is significantly lower in Japan than in Western countries. The purpose of this study was to investigate differences in circulating microRNA (miRNA) levels related to IHD in Austrians and Japanese. Participants were middle-aged healthy male Austrians (n = 20) and Japanese (n = 20). Total miRNAs in serum from each participant were analyzed using the 3D-Gene miRNA Oligo chip. Twenty-one miRNAs, previously reported as associated with IHD, were compared between Austrians and Japanese. The expression levels of miR-106a-5p, miR-135a-3p, miR-150-3p, miR-16-5p, miR-17-5p. miR-191-5p, miR-320b, miR-451a, miR-486-5p, miR-663b, and miR-92a-3p were significantly higher, while the miR-2861 expression level was significantly lower in Austrians as compared to Japanese. Both in Austrians and Japanese, there were significant positive correlations between serum expression levels of each pair of the above miRNAs except for miR-2861. The expression level of miR-2861 showed significant positive correlations with the expression levels of miR-106a-5p, miR-150-3p, miR-17-5p, miR-486-5p, miR-663b and miR-92a-3p in Austrians but not in Japanese. In pathway analysis, proinflammatory cytokine production in foam cells and collagen synthesis in vascular smooth muscle cells were associated with differentially expressed miRNAs. Difference in miRNA levels may contribute to lower cardiovascular risk in Japan than in Western countries.

## Introduction

The rate of mortality from ischemic heart disease (IHD) represented by myocardial infarction in Japan is lower than those in Western countries including Austria and the US: Age-standardized rates of death per 100,000 (ASDRs) from IHD in both sexes in 2015 were 141.6 in Austria, 71.9 in Japan and 133.6 in the USA^1^. According to the recent national statistics, ASDRs from acute myocardial infarction in 2018 were 61.2 and 13.9 in Austrian and Japanese men, respectively^2,3^. The ASDR from IHD in France is also much lower (ASDR in both sexes, 70.9 in 2014) than those in other European countries^1^. This phenomenon is known as the French paradox, which is attributed to high consumption of red wine containing antioxidant polyphenol compounds by French people^4^. Similarly, the IHD mortality rate is much lower in Japan than in Western countries despite the fact that the percentages of Japanese who smoke and have hypertension, major risk factors for IHD, are relatively high in Japan^5^. This phenomenon is called the Japanese paradox^6^, but its cause remains to be clarified.

Dyslipidemia, especially high LDL cholesterolemia and low HDL cholesterolemia, is the most important risk factor for ischemic heart disease. However, in Japan, lipid intake from foods has recently been increasing, resulting in an increase of the average blood cholesterol level to a level comparable to that in Americans^6^. Thus, the great difference between the IHD mortality rates in Japanese and Americans is unlikely to be due to a difference in blood cholesterol levels. A family history of IHD is also known to be a risk factor for IHD, and heredity is thus suspected to be involved in the pathogenesis of IHD^7^. According to the results of a recent genome-wide association study on IHD-related genes, there are single nucleotide polymorphisms (SNPs) of genes associated with IHD. However, in most cases of SNPs, the odds ratio of each SNP for IHD was relatively low (≤ 1.3). Among the candidates of SNPs, short arm 21 locus on chromosome 9 (9q21) showed the strongest association with IHD^8,9^. However, the frequencies of the risk allele of 9q21 SNP were reported to be comparable in Japanese and Caucasians^10^. Thus, genes explaining the racial difference in mortality by IHD have not been found so far. Therefore, the prominent difference between IHD mortality rates in Japan and Western countries cannot be explained by differences in SNPs of IHD-related genes.

MicroRNAs (miRNAs) are non-coding single-strand RNAs consisting of 20–25 bases and bind complementarily to mRNAs of the targeted genes, resulting in suppression of their translation. More than 30% of the protein-coding regions in a gene are thought to be targeted by miRNA. Modulation of protein expression by miRNA is involved in the pathogenesis of a variety of diseases including cancer and cardiovascular disease. Various miRNAs that are involved in atherosclerosis and angiogenesis have recently been determined. The miR-143/-145 cluster is essential for differentiation of vascular smooth muscle cells and determines their phenotype switching^11^. miR-126 is involved in maintaining homeostasis of vascular endothelial cells through inhibiting angiogenesis, proliferation and migration, and supporting vascular integrity^12^.

miRNAs are secreted from cells and circulating miRNAs are now being focused on as new biomarkers for various diseases. There are various miRNAs that are reportedly altered in the blood of patients with IHD^13–15^. However, racial and/or ethnic differences in IHD-related miRNAs remain unknown. In this study, we focused on circulating miRNAs that have been reported to be associated with IHD and compared them in Austrians (as representative people in Western countries) and Japanese in order to investigate their possible contribution to the Japanese paradox regarding mortality by IHD.

## Results

### Characteristic of the participants

Table 1 shows a comparison of each variable in the Austrian and Japanese groups. Age was not significantly different between the groups. Height was significantly taller and body weight was significantly larger in Austrians than in Japanese. Body mass index (BMI) was significantly higher in Austrians than in Japanese. Systolic blood pressure, diastolic blood pressure and pulse pressure were not significantly different between Austrians and Japanese. Fasting blood sugar level was significantly higher in Japanese than in Austrians, while triglycerides were not significantly different between the two groups. HDL cholesterol was significantly lower and LDL cholesterol was significantly higher in Austrians than in Japanese.

**Table 1:** Characteristics of the participants.

	Austrians	Japanese	*p* value
Number	20	20	–
Age (years)	49.9 (6.3)	48.7 (6.4)	0.551
Height (cm)	180.3 (5.7)	173.2 (4.5)	0.000
Body weight (kg)	83.7 (9.1)	70.0 (10.6)	0.000
Body mass index (kg/m^2^)	25.7 (1.9)	23.3 (3.1)	0.006
Systolic BP (mmHg)	123.3 (10.1)	123.4 (11.8)	0.977
Diastolic BP (mmHg)	80.4 (5.3)	79.5 (11.3)	0.736
Pulse pressure (mmHg)	42.9 (7.4)	43.9 (8.9)	0.686
Fasting sugar (mg/dl)	78.8 (9.8)	97.7 (7.7)	0.000
Triglycerides (mg/dl)^a^	84 (61, 135)	94 (60, 141)	0.882
HDL cholesterol (mg/dl)	47.3 (14.8)	61.6 (15.8)	0.005
LDL cholesterol (mg/dl)	145.7 (30.4)	124.3 (34.0)	0.042

Means with standard deviations of the variables except for triglycerides are shown.

*BP* blood pressure. Probability (*p*) values for differences between Austrians and Japanese are shown.

^a^Levels of triglycerides are shown as medians with interquartile ranges.

### Comparison in Austrians and Japanese of serum levels of each miRNA expression that were reported to be associated with IHD

Among 21 circulating miRNAs that have been reported as associated with IHD^16–34^, significant differences between Austrians and Japanese were detected in expression levels of 12 miRNAs in serum (Table 2). The expression levels of miR-106a-5p, miR-135a-3p, miR-150-3p, miR-16-5p, miR-17-5p, miR-191-5p, miR-320b, miR-451a, miR-486-5p, miR-663b and miR-92a-3p were significantly higher in Austrians than in Japanese, while the expression level of miR-2861 was significantly lower in Austrians than in Japanese (Table 2). miR-135a-3p, miR-16-5p, miR-451a, miR-486-5p and miR-92a-3p showed a relatively high fold change (≥ 1.98) in the mean value of each miRNA intensity of Austrians versus Japanese (Table 2, Supplementary Figs. 2, 2).

**Table 2:** Ischemic heart disease-related miRNAs that showed significant differences between Austrians and Japanese.

miRNA	Intensity^a^	Fold change^b^	*q* value	Association^c^	References
106a-5p	5.70 ± 0.92 5.06 ± 0.64	1.55	0.014	Inverse	^16^
135a-3p	7.48 ± 0.87 6.48 ± 0.54	1.99	< 0.001	Positive	^17^
150-3p	4.75 ± 0.39 4.29 ± 0.25	1.65	< 0.001	Positive	^18–20^
16-5p	8.04 ± 1.16 6.61 ± 0.69	2.69	< 0.001	Inverse	^21^
17-5p	5.96 ± 0.90 5.24 ± 0.60	1.63	0.005	Positive Inverse	^22^ ^23^
191-5p	5.11 ± 0.63 4.28 ± 0.54	1.77	< 0.001	Positive Inverse	^24^ ^19^
2,861	12.63 ± 0.48 12.89 ± 0.27	− 1.21	0.002	Positive	^24^
320b	5.32 ± 0.60 4.96 ± 0.31	1.28	0.004	Inverse	^25^
451a	11.13 ± 1.25 9.53 ± 0.80	3.03	< 0.001	Positive	^26^
486-5p	8.65 ± 1.05 7.40 ± 0.55	2.38	< 0.001	Positive	^20,27^
663b	8.07 ± 0.41 7.48 ± 0.30	1.50	< 0.001	Positive	^28^
92a-3p	8.00 ± 0.94 7.01 ± 0.48	1.98	< 0.001	Positive Inverse	^26,27^ ^23^

^a^Means with standard deviations of log2-transformed intensities of each miRNA in Austrians (upper) and Japanese (lower).

^b^Fold change in each mean miRNA intensity of Austrians versus Japanese.

^c^Positive or inverse association between each miRNA and ischemic heart disease reported previously.

Table 3 shows the results of serum miRNA expression levels that were found not to be significantly different in Austrians and Japanese. In previous studies, miR-1254, miR-197-3p, miR-221-3p, miR-223-3p, miR-23a-3p, miR-30d-5p, miR-3135b and miR-328-3p were reported to be positively associated with IHD, while miR-26a-5p was reported to be inversely associated with IHD. All of the above miRNA expression levels were not significantly different in Austrians and Japanese.

**Table 3:** Ischemic heart disease-related miRNAs that showed no significant differences between Austrians and Japanese.

miRNA	Intensity^a^	Fold change^b^	*q* value	Association^c^	References
1,254	5.81 ± 0.40 5.80 ± 0.24	1.00	0.431	Positive	^29^
197-3p	4.74 ± 0.38 4.67 ± 0.28	1.04	0.254	Positive	^30^
221-3p	4.95 ± 0.60 4.95 ± 0.67	1.00	0.431	Positive	^31^
223-3p	6.63 ± 0.81 6.97 ± 0.76	− 1.27	0.138	Positive	^30^
23a-3p	6.02 ± 0.61 6.08 ± 0.51	− 1.04	0.372	Positive	^32^
26a-5p	5.08 ± 0.83 4.99 ± 0.82	1.06	0.378	Inverse	^16,19^
30d-5p	5.66 ± 0.86 5.39 ± 0.46	1.21	0.111	Positive	^33^
3135b	9.05 ± 0.34 9.05 ± 0.25	1.00	0.416	Positive	^24^
328-3p	4.70 ± 0.41 4.58 ± 0.26	1.08	0.071	Positive	^34^

^a^Means with standard deviations of log2-transformed intensities of each miRNA in Austrians (upper) and Japanese (lower).

^b^Fold change in each mean miRNA intensity of Austrians versus Japanese.

^c^Positive or inverse association between each miRNA and ischemic heart disease reported previously.

### Correlations between serum expression levels of each pair of miRNAs that showed significant differences in Austrians and Japanese

**Figure 1:**
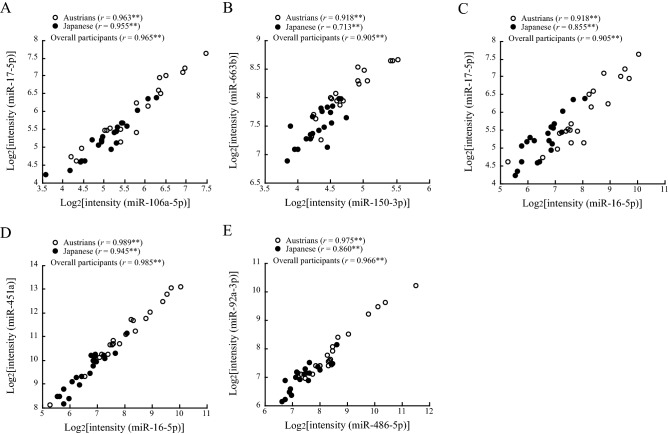
Correlations between each pair of miRNAs. Pearson’s correlation coefficients in Austrians, Japanese and overall participants are shown in the figures. Asterisks denote significant correlations (**p < 0.01).

Table 4 shows the correlations between expression levels of each pair of miRNAs in Austrians (*upper line*) and Japanese (*lower line*) that were found to be significantly different in the two groups (Table 2). Both in Austrians and Japanese, strong positive correlations (Pearson’s correlation coefficient ≥ 0.7) were found between intensity levels of the following 13 pairs: miR-106a-5p and miR-16-5p, miR-106a-5p and miR-17-5p, miR-106a-5p and miR-451a, miR-106a-5p and miR-92a-3p, miR-135a-3p and miR-150-3p, miR-150-3p and miR-663b, miR-16-5p and miR-17-5p, miR-16-5p and miR-451a, miR-16-5p and miR-92a-3p, miR-17-5p and miR-451a, miR-17-5p and miR-92a-3p, miR-451a and miR-92a-3p, and miR-486-5p and miR-92a-3p. Among these pairs, very strong correlations (Pearson’s correlation coefficient ≥ 0.9 in overall participants) were found between the following 5 pairs: miR-106a-5p and miR-17-5p, miR-150-3p and miR-663b, miR-16-5p and miR-17-5p, miR-16-5p and miR-451a, and miR-486-5p and miR-92a-3p (Table 4, Fig. 1).

**Table 4:** Correlations for each pair of microRNAs that showed significant differences between Austrians and Japanese.

	106a-5p	135a-3p	150-3p	16-5p	17-5p	191-5p	2,861	320b	451a	486-5p	663b	92a-3p
106a-5p	1.000 1.000											
135a-3p	− 0.054 0.384	1.000 1.000										
150-3p	0.266 0.382	0.729** 0.781**	1.000 1.000									
16-5p	0.879** 0.897**	0.278 0.262	0.414 0.285	1.000 1.000								
17-5p	0.963** 0.955**	0.128 0.345	0.427 0.370	0.918** 0.855**	1.000 1.000							
191-5p	0.644** 0.734**	0.205 0.452*	0.333 0.443	0.641** 0.671**	0.627** 0.751**	1.000 1.000						
2,861	0.496* − 0.286	0.405 − 0.042	0.747** 0.015	0.406 − 0.159	0.571** − 0.225	0.301 − 0.058	1.000 1.000					
320b	0.249 0.149	0.267 0.675**	0.652** 0.453*	0.252 − 0.105	0.342 0.061	0.378 0.122	0.682** − 0.324	1.000 1.000				
451a	0.877** 0.805**	0.260 0.241	0.363 0.261	0.989** 0.945**	0.904** 0.762**	0.590** 0.470*	0.372 − 0.063	0.178 − 0.111	1.000 1.000			
486-5p	0.640** 0.638**	0.592** 0.525*	0.762** 0.459*	0.747** 0.621**	0.735** 0.589**	0.531* 0.359	0.751** − 0.282	0.469* 0.360	0.720** 0.653**	1.000 1.000		
663b	0.236 0.352	0.709** 0.621**	0.918** 0.713**	0.370 0.224	0.396 0.403	0.312 0.514*	0.782** − 0.068	0.726** 0.457*	0.321 0.148	0.706** 0.357	1.000 1.000	
92a-3p	0.719** 0.871**	0.572** 0.579**	0.771** 0.582**	0.825** 0.799**	0.814** 0.874**	0.640** 0.703**	0.719** − 0.171	0.476* 0.300	0.796** 0.749**	0.975** 0.860**	0.708** 0.533*	1.000 1.000

Upper, Pearson’s correlation coefficients in Austrians; lower, Pearson’s correlation coefficients in Japanese. Asterisks denote significant correlations (*p < 0.05; **p < 0.01).

**Figure 2:**
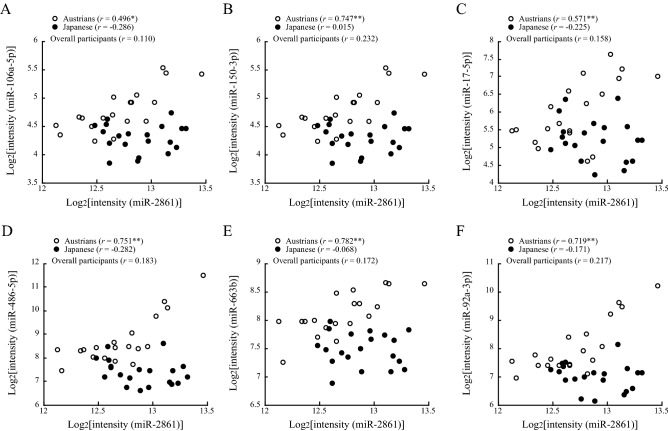
Correlations of miR-2861 with miR-106a-5p, miR-150-3p, miR-17-5p, miR-486-5p, miR-663b and miR-92a-3p. Pearson’s correlation coefficients in Austrians, Japanese and overall participants are shown in the figures. Asterisks denote significant correlations (*p < 0.05; **p < 0.01). An outlier of miR-2861 (log2[2341.8] = 11.19) was removed in the figure.

In Austrians, miR-2861 levels showed significant positive correlations with expression levels of miR-106a-5p, miR-150-3p, miR-17-5p, miR-486-5p, miR-663b and miR-92a-3p. On the other hand, in Japanese, none of the miRNAs examined showed a significant correlation with miR-2861 (Table 4, Fig. 2).

### Principal components of miRNAs that showed significant differences between Austrians and Japanese

The results of principal component analysis for serum expression levels of miRNAs are shown in Table 5. The main components were created for miRNAs showing associations with IHD in previous studies and significant differences between the two countries in this study (Table 2). Two major principal components of miRNAs were extracted in the Austrian subjects, while there were three principal components in the Japanese subjects. Both in the Austrian and Japanese groups, the miRNAs mainly constituting the first principal component were miR-106a-5p, miR-16-5p, miR-17-5p, miR-191-5p, miR-451a and miR-92a-3p, and the miRNAs mainly constituting the second principal component were miR-135a-3p, miR-150-3p, miR-320b and miR-663b. miR-2861 was included in the miRNAs mainly constituting the second principal component in the Austrian group, while miR-2861 constituted an independent component of the third principal component in the Japanese group.

**Table 5:** Eigenvalues of each miRNA determined by principal component analysis in Austrians and Japanese.

miRNA	Austrians		Japanese		
	The first PC (CPVE: 61.3%)	The second PC (CPVE: 81.6%)	The first PC 1 (CPVE: 53.5%)	The second PC ‘(CPVE: 72.7%)	The third PC (CPVE: 82.7%)
106a-5p	1.060	− 0.220	0.931	0.012	0.132
135a-3p	− 0.218	0.865	0.026	0.909	− 0.021
150-3p	− 0.042	0.977	0.081	0.899	− 0.204
16-5p	0.986	− 0.043	1.030	− 0.167	− 0.012
17-5p	0.981	− 0.023	0.928	0.023	0.035
191-5p	0.709	0.034	0.653	0.319	− 0.224
2,861	0.154	0.760	− 0.128	0.211	− 0.939
320b	− 0.056	0.763	− 0.335	0.745	0.460
451a	1.001	− 0.102	0.978	− 0.192	− 0.027
486-5p	0.513	0.567	0.579	0.230	0.327
663b	− 0.102	1.011	0.050	0.851	− 0.152
92a-3p	0.620	0.502	0.780	0.321	0.066

Eigenvalues of twelve miRNAs for each principal component are shown. PC, principal component; CPVE, cumulative percentage of variance explained by principal components. The eigenvalues of major miRNAs constituting each principal component are indicated with underlines.

### Atherosclerosis-associated pathways predicted to involve the miRNAs that showed significant differences between Austrians and Japanese

**Figure 3:**
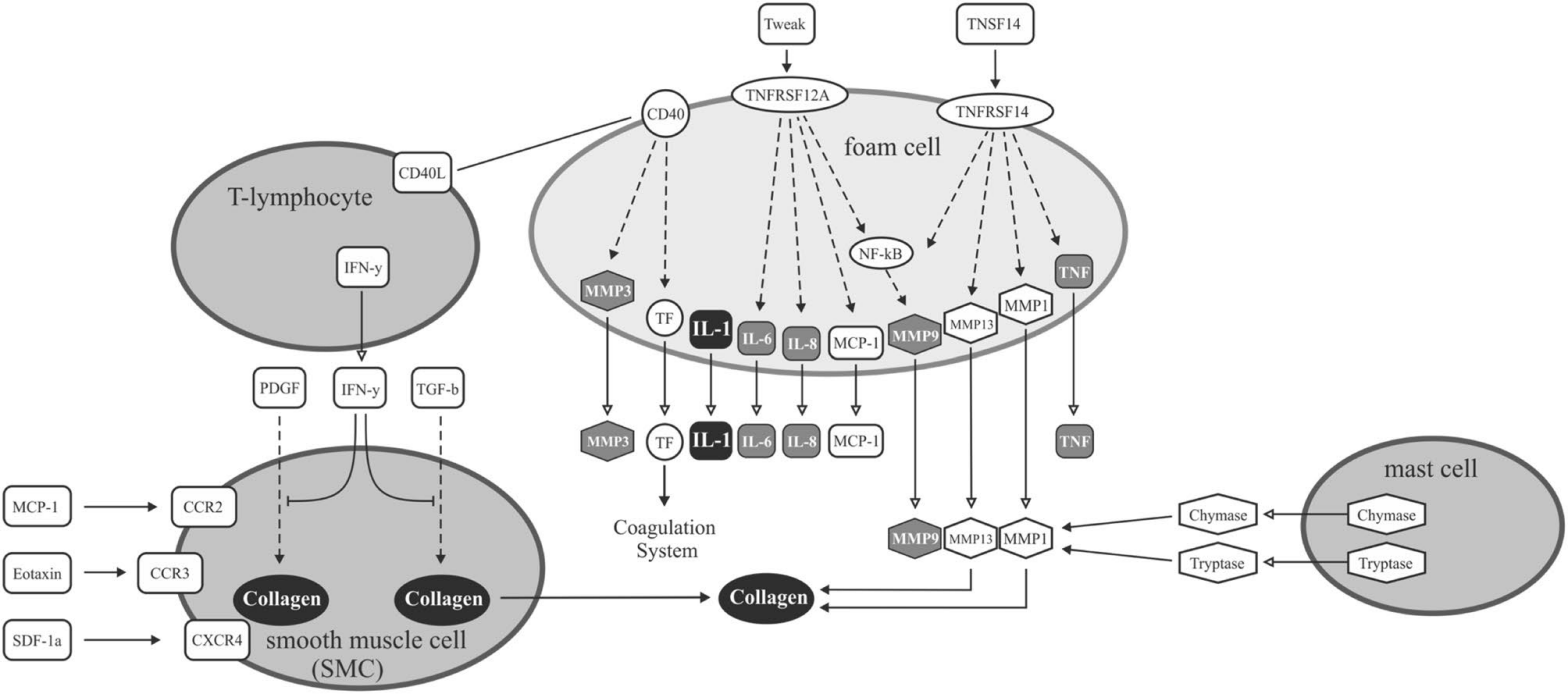
Overlap of identified miRNA targets with pathways related to the pathogenesis of atherosclerosis. Highlighted are the molecules that showed significant associations in Ingenuity Pathway Analysis with the 392 miRNAs found to be significantly different between Austrians and Japanese in this study.

We entered all of the miRNAs (n = 392), which showed significant differences between the two countries in microarray expression analysis, in Ingenuity Pathway Analysis (IPA). Proinflammatory cytokines (IL-1, IL-6, IL-8 and TNF) and matrix metalloproteinase (MMP)-3 and MMP-9 in foam cells and collagen in smooth muscle cells on arterial wall were found to be associated with these miRNAs (Fig. 3).

### Correlations between serum levels of each pair of miRNAs that showed no significant difference in Austrians and Japanese

Table 6 shows the correlations between intensity levels of each pair of miRNAs in Austrians (*upper line*) and Japanese (*lower line*) that were found not to be significantly different in the two groups (Table 3). Both in Austrians and Japanese, strong positive correlations (Pearson’s correlation coefficient ≥ 0.7) were found between only the following 3 pairs: miR-221-3p and miR-23a-3p, miR-223-3p and miR-23a-3p, and miR-23a-3p and miR-26a-5p (Table 6). In Austrians, there were relatively strong positive correlations (Pearson’s correlation coefficient ≥ 0.8) between miR-328-3p and miR-30d-5p and between miR-328-3p and miR-3135b, while the correlation coefficients of these miRNA pairs were not significant in Japanese (Table 6).

**Table 6:** Correlations for each pair of microRNAs that showed no significant differences between Austrians and Japanese.

	1,254	197-3p	221-3p	223-3p	23a-3p	26a-5p	30d-5p	3135b	328-3p
1,254	1.000 1.000								
197-3p	0.391 0.119	1.000 1.000							
221-3p	− 0.359 − 0.196	0.292 0.088	1.000 1.000						
223-3p	− 0.350 − 0.238	0.051 0.212	0.757** 0.252	1.000 1.000					
23a-3p	− 0.323 − 0.322	0.083 0.142	0.819** 0.767**	0.906** 0.747**	1.000 1.000				
26a-5p	− 0.278 − 0.092	0.000 0.013	0.775** 0.592**	0.872** 0.631**	0.863** 0.724**	1.000 1.000			
30d-5p	0.361 − 0.072	0.741** 0.237	0.317 0.882**	0.109 0.257	0.015 0.671**	− 0.011 0.508*	1.000 1.000		
3135b	0.588** − 0.167	0.556** 0.153	0.413 0.549*	0.246 0.645**	0.273 0.634**	0.283 0.663**	0.736** 0.476*	1.000 1.000	
328-3p	0.637** 0.300	0.714** 0.391	0.128 − 0.168	0.000 − 0.062	0.007 − 0.238	− 0.033 − 0.126	0.812** 0.142	0.800** 0.056	1.000 1.000

Upper, Pearson’s correlation coefficients in Austrians; lower, Pearson’s correlation coefficients in Japanese. Asterisks denote significant correlations (*p < 0.05; **p < 0.01).

## Discussion

This is to our knowledge the first study in which ethnic differences in circulating IHD-associated miRNA expression levels were investigated. Twelve miRNAs that have been reported as associated with IHD displayed significantly different levels of expression in Austrians and Japanese. Notably, miR-135a-3p, miR-16-5p, miR-451a, miR-486-5p and miR-92a-3p levels were prominently higher (1.98 fold or more) in Austrians than in Japanese (Table 2). Since circulating miR-135a, miR-451a and miR-486-5p are positively associated with IHD, these miRNAs are candidates for a novel mechanism to contribute to the different incidence between Japan and Western countries. Among the expression levels of miRNAs showing significant differences in Austrians and Japanese, only the expression level of miR-2861 was lower in the Austrian group than in the Japanese group, while miR-2861 was reported to be positively associated with coronary artery calcification^24^. Although miR-106a-5p and miR-16-5p showed inverse associations with IHD in previous studies^16,21^, their expression levels were significantly higher in Austrians than in Japanese in the present study. Therefore, miR-106a-5p, miR-16-5p and miR-2861 are unlikely related to the difference between the IHD mortality rates in Japan and Western countries. The Austrian group showed significantly higher miR-17-5p, miR-191-5p and miR-92a-3p expression levels than the levels in the Japanese group, but results regarding the relations of these circulating miRNAs with IHD were controversial in previous studies^19,22–24,26,27^, and thus the significance of miR-17-5p, miR-191-5p and miR-92a-3p in IHD remains to be clarified.

While the participants of the present study were healthy and were not taking any medications, most of the miRNAs examined in this study (Tables 2, 3) displayed divergent levels in patients with established coronary artery disease versus controls in previous studies. Therefore, future studies are needed to clarify the significance of the differences between Austrians and Japanese regarding circulating miRNA levels in healthy individuals to predict future IHD. There has been, to our knowledge, only one study on circulating miRNAs for predicting IHD. Bye et al. evaluated 179 miRNAs by using quantitative PCR (qPCR) and proposed 5 miRNAs, miR-106a-5p, miR-424-5p, let-7g-5p, miR-144-3p and miR-660-5p, as predictors of future myocardial infarction in healthy men and women^16^. In the present study, miR-106a-5p among the above-mentioned five miRNAs was available for comparison, while the intensities of the other miRNAs were too low to perform accurate analysis. miR-106a-5p was reported to be inversely associated with acute myocardial infarction^16^, but its level was significantly higher (fold change: 1.55) in Austrians than in Japanese (Table 2). Therefore, circulating miR-106a-5p is not suggested as a candidate for explaining the Japanese paradox by our study.

Here we report three miRNAs, miR-135a, miR-451a and miR-486-5p, of which the blood expression levels have been reported to be positively associated with IHD^17,20,26,27^, to be prominently higher (fold change: miR-135a, 1.99; miR-451a, 3.03; miR-486-5p, 2.38) in Austrians than in Japanese. Regarding their pathophysiological significance, miR-135a has been reported to be involved in apoptosis of rat cardiomyoblast cells by down-regulating Bcl-2^35^. miR-486 has been suggested as related to cardiac hypertrophy: miR-486 up-regulation contributes to the activation of Bcl-2-related mitochondrial apoptotic pathways, thereby exhibiting an anti-apoptotic function in cardiomyocytes^36^. From these findings in experimental studies, miR-135a and miR-486 are thought to work oppositely regarding the Bcl-2-regulated apoptotic pathway in remodeling post myocardial infarction. On the other hand, both miR-135a and miR-486 in blood showed positive associations with IHD in epidemiological studies^17^,^20^,^27^ and were significantly higher in Austrians than in Japanese in the present study. miR-451 was reported to be highly expressed in cardiac myocytes and to promote lipotoxicity and cardiac hypertrophy in obese mice^37^. It remains to be clarified whether and how changes in circulating levels of each miRNA in patients with IHD mechanistically impact disease progression through target mRNA regulation. Thus, future studies are needed to elucidate the pathophysiological significance of the racial differences in these circulating miRNAs.

Both in Austrians and Japanese, there were strong positive correlations between the 13 pairs of miRNAs that showed significant differences in the Austrian and Japanese groups in this study (Table 2). The correlations were generally stronger in the pairs of these miRNAs (Table 4) than in the pairs of the miRNAs that showed no significant differences in the Austrian and Japanese groups (Table 6). Therefore, we speculated that a common factor or mechanism exists in the associations of IHD with the miRNAs that were significantly different in the Austrians and Japanese.

It is reasonable to expect strong correlations between miR-16-5p and miR-451a and between miR-486-5p and miR-92a-3p since these miRNAs are known to bind to argonaute (AGO)2 in erythrocytes^38^, from which AGO2-bound miRNAs are secreted into blood. Thus, one might consider that hemolysis causes the differences in the Austrians and Japanese groups. However, this possibility is unlikely because levels of miR-144-3p, which is also known to be an AGO2-bound miRNA in erythrocytes^38^, were not significantly different between the two groups, and serum expression levels of let-7f-5p and miR-25-3p, other AGO2-bound miRNAs in erythrocytes^38^, were too low to perform analysis for comparison in the present study.

Interestingly, miR-2861 was significantly correlated with miR-106a-5p, miR-150-3p, miR-17-5p, miR-486-5p, miR-663b and miR-92a-3p in Austrians, while none of the miRNAs examined showed a significant correlation with miR-2861 in Japanese (Table 4, Fig. 2). The above findings on miR-2861 obtained by regression analysis agree with the result of principal component analysis that miR-2861 was included in the second principal component in the Austrian group, while miR-2861 alone was included independently in the third principal component in the Japanese group. Target genes of miR-2861 were predicted in silico as shown in Supplementary Table 1^39^. Validation of these targets by qPCR in the vessel wall where atherosclerosis progresses will be an interesting subject for future studies using tissue specimens. miR-2861 is known to enhance arterial calcification and osteoblastic formation of vascular smooth muscle cells^40^. Therefore, the process of calcification in the context of atherosclerosis may be differently modified through miR-2861 in Austrians and Japanese. miR-2861-binding site exists in the coding region of Hdac5 (histone deacetylase 5) (605315), which is involved in bone formation by enhancing Runx2 (600211) degradation. Overexpression of pre-miR-2861 in ST2 cells reduced endogenous Hdac5 protein and increased endogenous Runx2 protein, but it had no effect on their mRNA levels. Silencing of miR2861 by injection of antago-miR-2861 reduced Runx2 protein expression, inhibited bone formation, and decreased bone mass in mice^41^.

miR-150 is known to be an inflammation-associated miRNA, to be highly expressed in immune cells, and to regulate monocyte migration and proinflammatory cytokine production^42^. miR-150 has been shown as involved in the pathogenesis of cardiovascular disease and to impair myocardial contractility and post-myocardial infarction remodeling^43–45^. miR-663b is a cancer-associated miRNA and has been shown to be associated with various types of cancer^46–49^. Circulating miR-663b level has also been reported to be higher in patients with acute myocardial infarction than in a cohort without it^28^. However, the reason for the strong correlation between miR-150-3p and miR-663b (Table 4, Fig. 1) remains unknown.

There were significant differences in LDL and HDL cholesterol levels between Austrians and Japanese (Table 1). Since dyslipidemia is a major risk factor for IHD, the difference in blood cholesterol levels may contribute to the difference in the incidence of IHD between the countries, although average LDL cholesterol level of Japanese has recently been increasing and is close to that of Americans^6^. Austrian subjects showed significantly lower fasting blood sugar levels than Japanese subjects (Table 1). We do not have a good explanation why blood sugar levels are different between Japanese and Austrians in our subject samples. Both cohorts were fasted overnight, and earlier dinner times in Austria may be an explanation, although this is speculative. This may be to some extent a chance finding as the number of subjects in our pilot study was limited. Interestingly, regarding cholesterol levels, we saw the opposite trends as aforementioned, which may be explained by nutritional differences between Austrians and Japanese. BMI was also significantly higher in Austrians than in Japanese (Table 1).

We are aware of the limitations of this study. Our study is a hypothesis-supporting exploratory study, and thus a validation study will be needed to confirm our findings in a large, independent and prospective cohort. As our study had a limited sample size and was a pilot study, we did only include male subjects to reduce variability. Although 2,565 miRNAs in total were evaluated by using the miRNA Oligo chip, only 821 miRNAs were available for comparison, and circulating levels of the other miRNAs, including miR-126-3p, miR-126-5p, miR-133a-3p, miR-134-5p, miR-1-3p, miR-145-5p, miR-150-5p, miR-208a-3p, miR-21-5p and miR-499a-5p, which were shown to be biomarkers for IHD in previous studies^13–15^, were too low to analyze for comparison in the present study. Thus, further studies in which miRNA levels are measured using qPCR are needed to determine whether there are racial differences in levels of the above known miRNAs that were not used for comparison in this study. Circulating levels of the miRNAs were directly evaluated without PCR amplification and compared in the two groups in this study. Therefore, blood levels of each miRNA evaluated in this study were high enough for analysis, meaning that these miRNAs may not only be useful for biomarkers but also have pathophysiological significance in IHD, which needs to be clarified in future studies. It is also a limitation of this study that validation of each miRNA expression by qPCR was not performed. Of note, in a previous study, using the same microarray (3D-Gene miRNA microarray, Toray Industries, Kamakura Japan), miR-451 expression levels that were demonstrated to be significantly different between the countries in this study were also validated by qPCR and confirmed to show similar results by microarray and qPCR^50^. In recent publications^51–53^, miRNA levels in patients with various types of cancer were evaluated only by the above microarray analysis without validation by qPCR. In addition, we used a strict threshold expression level for detection of each miRNA as mentioned in the Methods section.

As other limitations of this study, the number of participants in this study (n = 40) was limited and all of them were men. Therefore, further studies in a larger population including female participants are required to confirm our findings. Most previous studies on the relationships between miRNAs and IHD were cross-sectional in their design, and the clinical implication of some miRNAs for IHD is controversial in previous studies (Table 2). We evaluated only one miRNA (miR-106a-5p) that was reported to be a predictive marker for acute myocardial infarction^16^. Since there have been few studies on miRNAs as biomarkers for predicting IHD, future prospective studies on the relationships between miRNAs and cardiovascular events are warranted.

Among twelve miRNAs, miR-135a, miR-486, miR-2861, and miR-150 are candidate miRNAs explaining the mechanisms for the difference in the incidence of IHD in Austrians and Japanese. miR-135a and miR-486 are involved in apoptosis of cardiomyocytes and cardiac hypertrophy^35,36^. miR-2861 regulates osteoblast differentiation and calcification^40^, which may suppress progression of atherosclerotic lesion. miR-150 regulates monocyte migration and proinflammatory cytokine production^42^ and might also regulate myocardial contractility^43–45^. In pathway analysis using all of differentially expressed 392 miRNAs, proinflammatory cytokines (IL-1, IL-6, IL-8 and TNF), MMP-3 and MMP-9 in foam cells and collagen in vascular smooth muscle cells were associated with differentially expressed miRNAs. However, precise mechanisms, effect size, and the combined effect of miRNA deregulation in relation to other factors (metabolic, genetic, etc.) are difficult to interpret and pin down on single molecules. Clearly, our study is a first step with no immediate clinical impact as many other factors (e.g., environmental, genetic) contribute to the net cardiovascular risk in a given population, and much bigger studies are needed to control for these factors. However, we believe that our insights pave the way to identify pathways and/or molecules that are targeted by respective miRNAs in more detail (e.g. inflammation) in future studies.

In conclusion, among the 21 IHD-associated miRNAs examined, the blood levels of 12 miRNAs were found to be significantly different in Austrians and Japanese: miR-135a-3p, miR-16-5p, miR-451a, miR-486-5p and miR-92a-3p showed relatively high fold changes (≥ 1.98) in each miRNA intensity of Austrians versus Japanese. Although we present here a pioneering pilot study, our findings may serve as a basis for future detailed investigations to identify the pathophysiological mechanism underlying the IHD Japanese paradox.

## Methods

### Participants

Participants were healthy male Austrians (n = 20) and Japanese (n = 20) who were not receiving any medications. Their mean ages were 49.9 and 48.7 years (*p* = 0.551), respectively. All of the Austrian participants were Caucasians and lived in Graz, and all of the Japanese participants were originally from Japan and worked in Nishinomiya, Sasayama or Yamagata. All of the participants were nonsmokers. The protocol of this study was approved by the Hyogo College of Medicine Ethics Committee (No. 3036 in 2018) and the Medical University of Graz Ethics Committee (27-166 ex 14/15). Written informed consent was provided by all of the participants. All methods were performed in accordance with the relevant guidelines and regulations.

### Blood sample collection

Blood was collected from each participant after overnight fasting, and serum was separated. Serum samples were kept frozen at -80 degrees until analyses of miRNAs and variables related to cardiovascular risk as described below.

### Measurement of variables related to cardiovascular disease

Height and body weight were measured, and BMI was calculated as weight in kilograms divided by the square of height in meters. Blood pressure was determined by using an automatic digital blood pressure device. Pulse pressure was calculated as the difference between systolic and diastolic blood pressures. Fasted blood was sampled from each subject, and serum triglycerides, LDL cholesterol, HDL cholesterol and glucose concentrations were measured by conventional enzymatic methods.

### RNA extraction and miRNA expression profiling

RNA was extracted from a serum sample (300 µl) using 3D-Gene RNA extraction reagent from a liquid sample kit (Toray Industries Inc., Kamakura, Japan) according to the manufacturer’s instructions as follows: 3D-GeneTM RNA extraction reagent (0.9 ml) was added to each serum sample (0.3 ml). After vortexing for 1 min and storing at room temperature for 3 min and on ice for 3 min, the aqueous phase was separated by centrifugation at 12,000×*g* for 10 min at 4 degrees and stored at room temperature for 3 min. Ethanol (1.5 volume) was added to the aqueous phase solution in a new prelubricated tube, which was pipetted up to 700 µl into an RNeasy Mini spin column (Qiagen, Tokyo, Japan) and centrifuged at 8,000×*g* for 15 s at room temperature. The flow-through was discarded and this step was repeated using the remainder of the sample. The column was washed with Buffer RWT and Buffer RPE and transferred to a new 1.7-ml prelubricated tube. RNase-free water (30 µl) was pipetted onto the column membrane and after storing for 1 min at room temperature, RNA was eluted by centrifugation at 8,000×*g* for 1 min. Extracted total RNA was checked by Bioanalyzer (Agilent, CA, USA). A representative result of RNA extraction by Bioanalyser 2100 is shown in Supplementary Fig. 3. Concentrations of RNA in solutions extracted from original samples were 0.179 ~ 0.493 ng/µl.

miRNA expression was analyzed using the 3D-Gene miRNA Oligo chip (TRT-XR520, Toray) and 3D-Gene miRNA labeling kit (TRT-XE211, Toray) as described previously^54^. Briefly, half volumes of labeled RNAs were hybridized onto a 3D-Gene miRNA Oligo chip (Toray), which was designed to detect sequences of multiple miRNAs, and the annotation and oligonucleotide sequences of the probes were conformed to the miRBase release 21, miRNA database (https://microrna.sanger.ac.uk/sequences/). After stringent washes, fluorescent signals were scanned with a 3D-Gene Scanner (Toray) and analyzed using 3D-Gene Extraction software (Toray). miRNA expression was normalized as follows. The raw data of each spot were substituted with a mean intensity of the background signal determined by signal intensities of all blank spots with signal intensity of the top and bottom 5% (out of 95% confidence intervals) being removed. Measurements of spots were considered to be valid when the signal intensities were greater than 2 standard deviations of the background signal intensity. The signal intensities of the valid spots were compared and a relative expression level of a given miRNA was calculated throughout the microarray experiments. Global normalization of the data was performed for each array, such that the median of the signal intensity was adjusted to 25. Intensity levels of 10 or higher were regarded to be high enough for analysis of comparison between the two country groups. Each miRNA level was compared after log2-transformation between the groups. Fold change in the mean value of each miRNA intensity of Austrians versus Japanese was calculated as the ratio of anti-log2 values of each mean of log2-transformed data.

### Selection of IHD-related miRNAs

A total of 2,565 miRNAs in serum were measured by using the miRNA Oligo chip. As a result, the intensities of 1744 miRNA were too low for reasonable quantitative comparison. Consequently, 821 miRNAs were available for comparison between the Austrian and Japanese groups. Raw data for 821 miRNAs that showed high enough expression levels in blood for statistical analysis and raw data for 392 miRNAs that showed significant differences between Austrians and Japanese are provided in Supplementary Table 2.

From previous reports including recent review articles^13–15^, we further chose the following 21 miRNAs that have been reported as associated with IHD^16–34^ and showed intensities in serum that were high enough for evaluation: miR-106a-5p, miR-1254, miR-135a-3p, miR-150-3p, miR-16-5p, miR-17-5p, miR-191-5p, miR-197-3p, miR-221-3p, miR-223-3p, miR-23a-3p, miR-26a-5p, miR-2861, miR-30d-5p, miR-3135b, miR-320b, miR-328-3p, miR-451a, miR-486-5p, miR-663b and miR-92a-3p. In this study, expression levels in serum of the above 21 miRNAs were compared between Austrians and Japanese.

### Bioinformatics pathway analysis

The resultant differentially expressed miRNAs between Austrians and Japanese were analyzed using Ingenuity Pathway Analysis (IPA) (Ingenuity Pathway Analysis: QIAGEN Inc. https://www.qiagenbioinformatics.com/products/ingenuitypathway-analysis)^55^. Putative miRNA targets were found using Ingenuity miRNA target filter for experimentally validated and putative predicted targets (high confidence level) through in silico analysis^56^. After linking miRNAs with mRNA target genes, mRNA target genes that differed between the countries were considered. The IPA miRNA-mRNA target link module derives information from TargetScan, a database containing miRNAs and their predicted target genes, along with prediction scores and the experimental confirmation from the literature^57^.

### Statistical analysis

Continuous variables are summarized as means with standard deviations or medians with interquartile ranges and were compared with the use of Welch’s corrected t-test or the Wilcoxon-Mann–Whitney test, as appropriate. For each of the tested miRNAs, on the basis of the observed distribution of *p* values, we estimated the positive false discovery rate (*q* value) according to the method of Storey et al.^58^. Associations among the miRNAs were explored with the use of Pearson’s correlation coefficients. Associations among the miRNAs with significant *q* values were also explored with the use of the principal component analysis with promax rotation. The intensity of each miRNA was transformed with the base-2 logarithm, that is, binary logarithm. All *p* values were two-sided, and *p* values less than a significance level of 0.05 was considered statistically significant. All *q* values were two-sided, with statistical significance determined by a false discovery rate of less than 0.05. Data were analyzed with the use of SPSS version 22.0 Armonk, NY, USA.

## Supplementary information

Supplementary Figures.

Supplementary Table 1.

Supplementary Table 2.
