# Supplementary material for: Blood levels of microRNAs associated with ischemic heart disease differ between Austrians and Japanese: a pilot study
Source: Sci Rep. 2020 Aug 12;10:13628. doi: 10.1038/s41598-020-69332-0 (PMC7423897; doi:10.1038/s41598-020-69332-0)
Supplement: Supplementary file 1 — Supplementary Figures. [file 41598_2020_69332_MOESM1_ESM.pdf]

Supplementary Figures 1 ~ 3

Title: Blood levels of microRNAs associated with ischemic heart disease differ between Austrians and Japanese: a pilot study

Authors: Ichiro Wakabayashi[1], Ryoji Eguchi[1], Yoko Sotoda[2], Dirk von Lewinski[3], Harald Sourij[4], Takashi Daimon[5], Klaus Groschner[6], Peter P. Rainer[3]

From:
[1]Department of Environmental and Preventive Medicine, Hyogo College of Medicine, Nishinomiya, Hyogo 663-8501, Japan
[2]Department of Cardiovascular Surgery, Yamagata Saisei Hospital, Yamagata 990-8545, Japan
[3]Division of Cardiology, Medical University of Graz, Auenbruggerplatz 15, 8036 Graz, Austria
[4]Division of Endocrinology and Metabolism, Medical University of Graz, Auenbruggerplatz 15, 8036 Graz, Austria
[5]Department of Biostatistics, Hyogo College of Medicine, Nishinomiya, Hyogo 663-8501, Japan
[6]Gottfried Schatz Research Center for Cell Signaling, Metabolism and Aging. Medical University of Graz. Neue Stiftingtalstrasse 6/D04, 8010, Graz, Austria

Corresponding author:
Ichiro Wakabayashi, MD & PhD
Department of Environmental and Preventive Medicine,
Hyogo College of Medicine,
Mukogawa-cho 1-1, Nishinomiya, Hyogo 663-8501, Japan
Tel: +81-798-45-6561; Fax: +81-798-45-6563
E-mail: wakabaya@hyo-med.ac.jp

Supplementary Figure 1

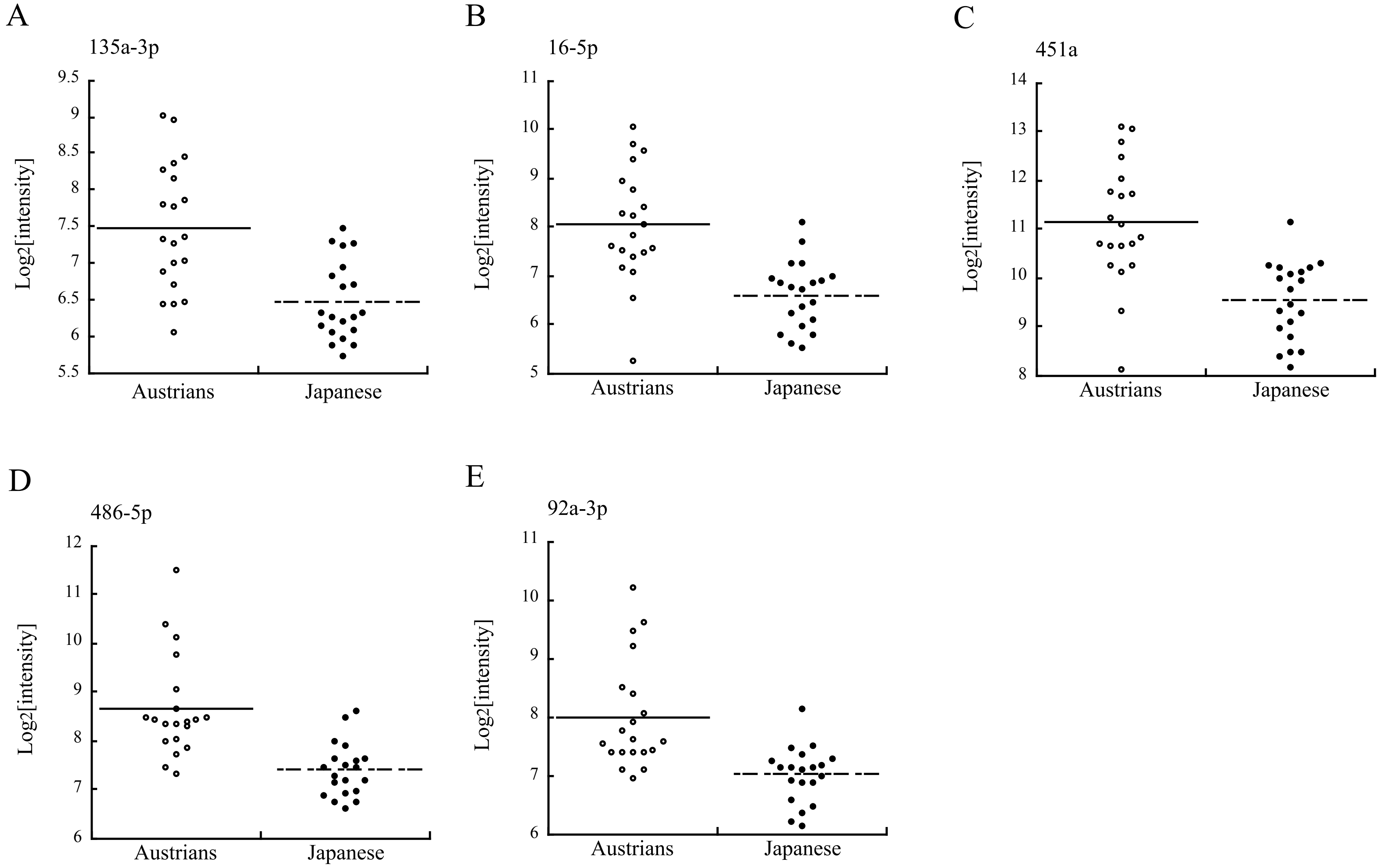

Supplementary Figure 1. Comparison of log2-transformed intensities of miR-135a-3p, miR-16-5p, miR-451a, miR-486-5p and miR-92a-3p in blood between Austrians and Japanese. Mean intensity levels are indicated by transverse bars.

Supplementary Figure 2

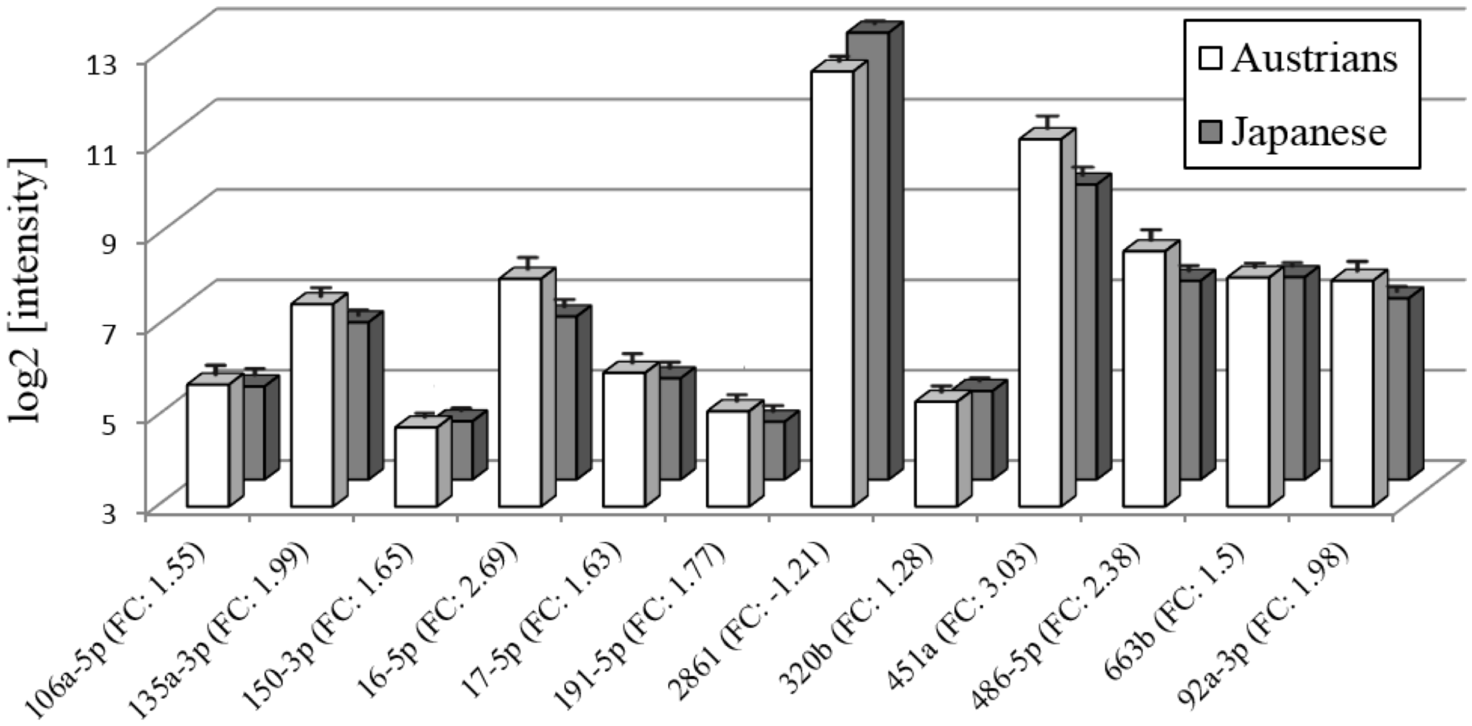

Supplementary figure 2.
Comparison of mean log2-transformed intensities of expression of the 12 miRNAs in blood between Austrians and Japanese. FC, fold change. Means with standard errors of the intensities are shown.

Supplementary Figure 3

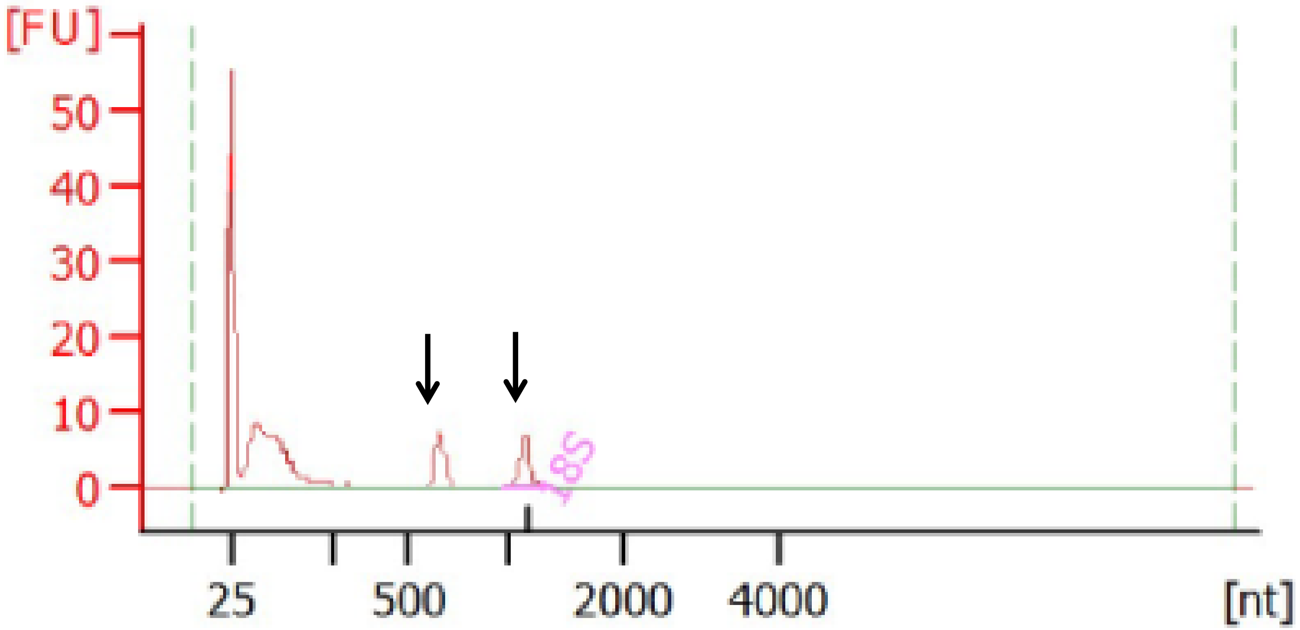

Supplementary Figure 3.
A representative electropherogram resulting from Agilent 2100 Bioanalyzer analysis on total RNA extracted from serum of the subjects. Arbitrary fluorescence units (FU) are plotted as a function of RNA size in nucleotides (nt). The arrows in the figure indicate peaks by RNA spike-in controls.
